# Genetically Encoded Fluorescent Biosensors for Biomedical Applications

**DOI:** 10.3390/biomedicines9111528

**Published:** 2021-10-24

**Authors:** Vera S. Ovechkina, Suren M. Zakian, Sergey P. Medvedev, Kamila R. Valetdinova

**Affiliations:** 1 The Federal Research Center Institute of Cytology and Genetics, The Siberian Branch of the Russian Academy of Sciences, 630090 Novosibirsk, Russia; vs_ovechkina@mail.ru (V.S.O.); zakian@bionet.nsc.ru (S.M.Z.); medvedev@bionet.nsc.ru (S.P.M.); 2 Department of Natural Sciences, Novosibirsk State University, 630090 Novosibirsk, Russia; 3 E.N. Meshalkin National Medical Research Center, Ministry of Health of the Russian Federation, 630055 Novosibirsk, Russia; 4 Institute of Chemical Biology and Fundamental Medicine, The Siberian Branch of the Russian Academy of Sciences, 630090 Novosibirsk, Russia

**Keywords:** genetically encoded fluorescent biosensors, fluorescent protein, drug screening

## Abstract

One of the challenges of modern biology and medicine is to visualize biomolecules in their natural environment, in real-time and in a non-invasive fashion, so as to gain insight into their physiological behavior and highlight alterations in pathological settings, which will enable to devise appropriate therapeutic strategies. Genetically encoded fluorescent biosensors constitute a class of imaging agents that enable visualization of biological processes and events directly in situ, preserving the native biological context and providing detailed insight into their localization and dynamics in cells. Real-time monitoring of drug action in a specific cellular compartment, organ, or tissue type; the ability to screen at the single-cell resolution; and the elimination of false-positive results caused by low drug bioavailability that is not detected by in vitro testing methods are a few of the obvious benefits of using genetically encoded fluorescent biosensors in drug screening. This review summarizes results of the studies that have been conducted in the last years toward the fabrication of genetically encoded fluorescent biosensors for biomedical applications with a comprehensive discussion on the challenges, future trends, and potential inputs needed for improving them.

## 1. Introduction

Genetically encoded fluorescent biosensors as a system include whole cells as well as cellular components that can be used for various analyte detection. Analytical abilities of the system are based on the cell’s natural capacity to perceive environmental signals and respond through changes in the level of biomolecules such as antibodies, enzymes, transcriptional factors, etc. A wide range of cell types can act as cell biosensors—from bacteria and lower eukaryotes, including fungi and yeast, to higher eukaryotes and their cell cultures. Meanwhile, prokaryotic cell-based biosensors reveal environmental fluctuations, human cell-based biosensors provide relevant context for testing drugs and can be used at the primary screening stage. Moreover, cell-based biosensors can be integrated into more complex in vivo and in vitro systems such as 3D structures and whole tissues, and reflect microenvironmental changes.

**Figure 1:**
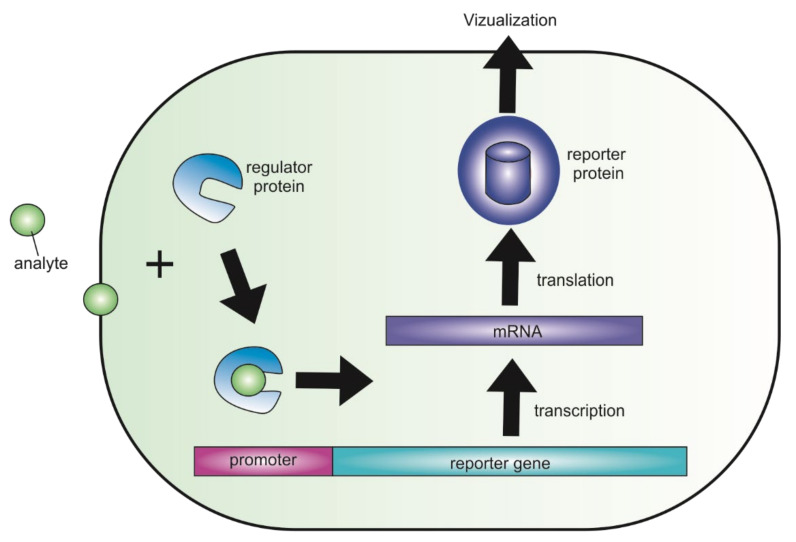
A schematic diagram that demonstrates how genetically encoded fluorescent biosensor works. An exogenous or endogenous analyte binds to a regulatory protein and changes gene expression. The altered expression can be further analyzed via analytical instruments (e.g., GFP can be detected with a fluorescent microscope).

Broadly, genetically encoded fluorescent biosensors are molecules that can be used to measure practically any biological process occurring in a cell in normal or pathological conditions. Changes in biochemical composition can be detected by various tests such as a microscope visualization. In common, genetically encoded fluorescent biosensor contains a sensing element that selectively binds an analyte molecule and a reporter unit that converts the interaction into detectable signals. Most often, the sensors are derived from genetically modified cells of the original organism, and produce chimeric proteins as a result of their vital activity, allowing to visualize the processes of biochemical activity of the cells (Figure 1). The DNA sequence of the chimeric proteins is often located downstream of a gene promoter, which can be activated by the target analyte. Fluorescent proteins (as red fluorescent protein, green fluorescent protein, etc.) are most commonly used as detectors in biosensors due to the ease of signal registration and wide dynamic range that enables the recording of small fluctuations in response to a target signal [1]. Furthermore, the lack of cytotoxicity is one of the major features of fluorescent proteins, so they do not affect the normal physiological processes of the cell and allow real-time monitoring at the same time.

Due to their structure, genetically encoded fluorescent biosensors have a wide range of detection capabilities. The main advantage of sensors over other types of detection is that in addition to qualitative detection of analytes, biosensors provide a rapid and sensitive assay for monitoring under natural physiological conditions. In common, genetically encoded fluorescent biosensors are cultivated in population, thus, the obtained data contain information registered via multiple cells and can be applied for cellular interactions observations. Thus, when interpreting the data, it should be taken into consideration that the obtained data on single cells reflect the cell behavior and its biochemical processes, which occur in the integrated cellular system [2].

Nowadays, genetically encoded fluorescent biosensors have become a promising and reliable tool for pollutant determination, drug screening, and investigation of toxicant presence. Relative to traditional methods of determining biological molecules in living systems, biosensors are cheaper, easier to use, more specific, faster, and capable of working in real-time. Therefore, their use in laboratory systems is rather efficient and convenient. In biomedical and clinical applications, genetically encoded fluorescent biosensors can respond to fluctuations in normal physiological activities of cells and tissues caused by external and inner stimuli. Genetically encoded fluorescent biosensors in combination with gene editing are accessible tools for visualization of signaling pathways and their disturbances, biomolecules and organelles interactions, cell culture monitoring, etc. Cells with electrogenic activity (neurons, myocytes, and pancreatic cells) have been particularly widely used in laboratory biomedical experiments. In these models, it is possible to measure the electrical response of cells when exposed to stimuli. The ability to use various cells as sensors combined with gene editing makes genetically encoded fluorescent biosensors a powerful biomedical tool with the potential to obtain data on various biochemical processes of interest and their abnormalities [3].

Here, we summarize current approaches in genetically encoded fluorescent biosensors construction and discuss their application in biomedicine as a platform for drug screening or detection of harmful biomolecules involved in disease development. We briefly describe the general structure of biosensors applied to detect biochemical fluctuations in the cell and consider different approaches to both the design of sensitive elements and the parts needed for imaging. This work also provides insight into the advantages, challenges, and prospects for the use of genetically encoded fluorescent biosensors in biomedical research.

## 2. General Characteristics of Genetically Encoded Fluorescent Biosensors

To begin with, in the review we define a genetically encoded fluorescent biosensor as a genetically modified cell with a chimeric reporter protein integrated into the biochemical circuit. The protein must be constructed to perceive intracellular or extracellular fluctuations and convert them into measurable readout. Thus, intracellular changes such as metabolites concentration, localization, conformation, and proteins’ activity can be visualized via analytical instruments. Hereby, the most appropriate and convenient way for conversion is fluorescence proteins utilization as a perfect device for monitoring the biochemical behavior of the cell. Fluorescence is fast and allows real-time detection as the light absorption and emission by a fluorophore is about nanoseconds. Moreover, the length of the emission light is less than cellular structures and enables fine and accurate spatial detection. In addition, modern genetically engineered fluorescent proteins are stable and present no cytotoxicity. All of the above advantages have made fluorescent proteins widely available for real-time imaging of biological pathways directly in living cells.

The molecular part of biosensors is derived into two general parts: a sensing element that perceives an analyte and a reporter that transforms emerged biochemical signals into a readable context that could be quantified. The sensing element is selected as involved in the molecular cascade of the event of interest, whereas the reporter element is a part of a fluorescent protein that is coupled to the sensing protein and integrated into the cell genome via gene engineering. The process under study can affect both the expression level of the sensing element and the conformational changes of the reporter element, and from these changes, it is possible to translate the information into a quantitative form [4].

**Figure 2:**
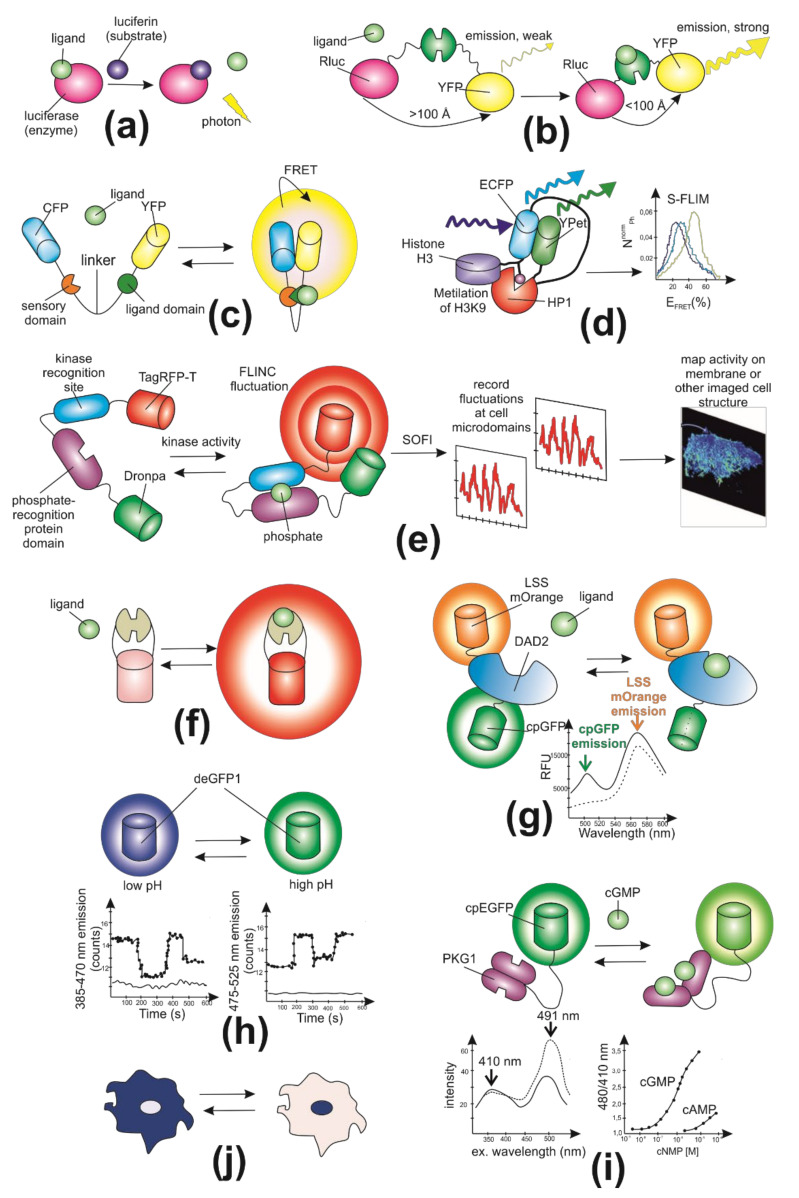
General types of biosensors grouped with principles of their readout mechanisms. (**a**) Schematic representation of bioluminescence intensity-based biosensor; (**b**) schematic representation of BRET biosensor; Rluc—*Renilla* luciferase, YFP—yellow fluorescent protein; (**c**) schematic representation of intensity-based FRET biosensor; CFP—cyan fluorescent protein; (**d**) schematic representation of the H3K9me3 chromatin compaction FLIM–FRET biosensor. The H3Kme3 biosensor is a construct of histone H3, enhanced cyan fluorescent protein (ECFP), Heterochromatic Protein 1 (HP1) and YPet. HP1 is responsible for the methylation of lysine 9 of the H3, which is a posttranslational modification known to induce heterochromatin formation, while ECFP and YPet are the FRET pair. Cells were transfected with the chromatin compaction biosensor (H3Kme3) and in hypo-osmolar or hyper-osmolar conditions to induce chromatin decompaction and compaction, respectively [11]. Phasor S-FLIM allows to compute the FRET efficiency with a four to five times increased number of photons compared to conventional FLIM, which results in much sharper images and distributions, revealing small differences in FRET efficiency at the single cell level. (**e**) Schematic of the FLINC-KAR biosensor design principle. FLINC phenomenon was characterized using Dronpa-TagRFP-T, where these two fluorescent proteins (FPs) are fused with A kinase activity domain and short flexible linker [9]. Phosphorylation of this biosensor leads to a change of fluorescence properties (decreasing the distance between Dronpa and TagRFP-T revealed a corresponding increase in TagRFP-T fluorescence fluctuations), thereby allowing us to monitor kinase activity by imaging a reporter, without labeling or disrupting the active kinase. The output of this FLINC-based biosensor is activity-dependent changes in the fluorescence fluctuations, which are readily quantified at super-resolution using photochromic Stochastic Optical Fluctuation Imaging (SOFI); (**f**) Schematic representation of intensity-based biosensor. (**g**) Schematic of the ratiometric DAD2 cpGFP (circularly permuted green fluorescent protein) biosensor design. Strigolactone (ligand) induced conformational change is propagated into cpGFP, modulating its fluorescence [12]. The C-terminal LSSmOrange fusion provides an internal fluorescent control; RFU—relative fluorescence units. (**h**) Schematic representation of emission ratiometric pH sensitive deGFP biosensor. Novel dual emission, pH-sensitive variants of GFP have been constructed and are suitable for ratiometric emission measurements in vivo [13]. (**i**) Schematic representation of excitation ratiometric FlincG biosensor. Fusion of regulatory fragments of PKG I (protein kinase I) to cpEGFP (circularly permuted enhanced green fluorescent protein) induces cGMP (cyclic 3′,5′-guanosine monophosphate) dependent changes in fluorescence emission intensity [14]. (**j**) Schematic representation of translocation-based biosensor.

According to biosensordb.ucsd.edu, there are ten general types of biosensors grouped with principles of their readout mechanisms: Bioluminescence intensity, BRET (bioluminescent resonance energy transfer), Intensity-based FRET (Förster resonance energy transfer), FLIM–FRET (time-resolved fluorescence microscopy-Förster resonance energy transfer), FLINC (fluorescence fluctuation increase by contact), intensity, ratiometric (with a reference fluorescent protein), excitation ratiometric, emission ratiometric, and translocation (Figure 2) [4]. The first group of biosensors is based on measuring directly the bioluminescence intensity. In bioluminescence, ligand binding causes an intramolecular reaction in which luciferin is oxidized, resulting in the emission of photons (Figure 2a). The photon emission rate is proportional to the amount of bound ligand. On the one hand, bioluminescent sensors offer increased sensitivity compared with fluorescent ones because the technique of visualization does not require incident radiation. Moreover, bioluminescent and fluorescent sensors have no spectral overlaps so can be applied in combination to control biological systems. On the other hand, bioluminescent sensors are mostly used in macroscopic imaging with low resolution due to the low-brightness nature of bioluminescence [5]. In BRET, the donor chromophore is luciferase (or one of its variants), and the acceptor is a fluorescent protein, for example, YFP (yellow fluorescent protein) (Figure 2b). Fluorescence resonance energy transfer (FRET) is a process of energy transfer between an excited molecular fluorophore (the donor) to another fluorophore (the acceptor) through intermolecular coupling [6] (Figure 2c). FRET-based sensors have made it possible to measure dynamic changes in the concentration of molecules of interest with high spatial and temporal resolution. Such sensors are chimeric proteins containing a fluorescent pair with overlapping excitation and emission spectra. Analyte binding changes the conformation of protein molecules, thus influencing the FRET efficiency [7]. FLIM-based FRET measurement (FLIM–FRET) consists of a fluorescent pair with well-separated emission spectra (Figure 2d). Thus, the combination of an enhanced green fluorescent protein (EGFP) and a red fluorescent protein (RFP) prevents fluorescent contamination. However, Förster distance is relatively short, and the range of proteins emission wavelength is wide. These disadvantages impose restrictions on multicolor imaging with additional dyes utilization [8]. FLINC-based biosensors could provide activity information in super-resolution (Figure 2e). For example, FLINC-based PKA (protein kinase A) biosensor allowed authors to resolve minute PKA activity microdomains on the plasma membrane of living cells and uncover the role of clustered anchoring proteins in organizing these activity microdomains [9]. Intensity-based biosensors work on the principle of increasing or decreasing the fluorescence intensity (Figure 2f). Ratiometric biosensors are widespread among genetically encoded fluorescent biosensors. The protein probes are sensitive to the physical and chemical parameters of the cell (e.g., pH, ions, and voltage) and reflect the fluctuations through a shift in its fluorescent emission (Figure 2g). As an example of pH-dependent sensors, the emission transformation depends on the state of protonation. Thus, the protonated form of the fluorescent protein can be detected using excitation at 405 nm, and deprotonated via excitation at 488 nm [10]. Ratiometric biosensors rely on a change in the fluorescence ratio between two emission wavelengths, rather than a fluorescence intensity change, and therefore are less dependent on biosensor concentration. Ratiometric probes, which have multiple excitation or emission maxima that show opposing changes in fluorescence excitation or emission in response to changes in analyte concentration, are potentially much more useful. Ratiometric measurements can reduce or eliminate distortions of data caused by photobleaching, indicator concentration, variable cell thickness, illumination stability, excitation path length, and nonuniform indicator distribution within cells or between groups of cells. For some applications intensity ratios have been preferred, and especially the ratio of emission intensity at two different excitation wavelengths (Figure 2i). This is because in the fluorescence microscope changing excitation wavelengths has little impact on the image quality, whereas changing the emission wavelength often affects the image quality. Moreover, changing the excitation wavelength can be done quickly using acousto-optic tunable filters to image rapidly changing specimens, whereas this is harder on the emission side. Currently, there are a large number of fluorescence microscopes capable of excitation ratiometric biosensor measurements in use. However, in some cases, emission ratiometric biosensors are used (Figure 2h). Translocation-based biosensors make it possible to track the movement of a fluorescent signal between cellular compartments (Figure 2j). These “moving sensors” are usually used for qualitative measurements because they are difficult to use for obtaining quantitative information.

## 3. Key Parameters in Biosensor Design

Given the variety of biosensors available, both chemical and genetically encoded, the choice of the optimal sensor requires a careful assessment of their biophysical properties, which are more in line with the needs of the experimenter. The most important parameters to consider when choosing a biosensor are the following

(1) Ease of use. One of the critical factors when choosing a biosensor is the availa-bility of the equipment needed for the measurements. For example, ratiometric biosensors based on FRET require sophisticated microscopic equipment to quickly (or simultaneously) collect data from two or more fluorescence channels. At the same time, for biosensors that measure fluorescence intensity, uninvolved instruments are needed to collect data. It is also necessary to consider the availability of the materials used. Finally, many luminescent biosensors require an additional step of adding a substrate such as coelenterazine.
(2) Sensitivity of a biosensor is the dynamic range, which is the ratio between the minimum and maximum values that the biosensor can detect. The sensitivity of the biosensor used is application-specific. For example, to measure constant concentrations of a molecule or ion, it is recommended to choose a sensor with a dissociation constant equal to or close to the expected concentration. An even more rigorous approach is to measure the concentration at rest using several biosensors, each of which has a slightly different dissociation constant value. If the goal is to measure a change in concentration or activity when the signal is expected to be weak, then the biosensor with the highest dynamic range and dissociation constant value, at which the concentration change will be within 5 dissociation constant, will be most sensitive.
(3) The detection limit of a sensor is defined often by the range in which the binding isotherm has near-linear characteristics, that is, between 10% and 90% saturation; often two orders of magnitude for a FRET sensor with a Hill coefficient of 1. Due to the dramatic intensity changes caused by ligand binding in fluorescent sensors, the dynamic range can be extended beyond the linear range. Signal-to-background ratio depends not only on sensor properties, but importantly on the background fluorescence in the specimen under investigation. Autofluorescence can differ substantially. Handling, stress conditions can trigger production of fluorigenic compounds.
(4) Selectivity and specificity are two important features to consider when developing or using existing biosensors. Selectivity and specificity are determined by the structure and conformational flexibility of a protein. The terms are often used interchangeably, but are best used for different aspects: specificity is defined as how restrictive a protein is in its choice of substrate (fewer vs. more substrates). Selectivity is defined by substrate properties and is a quantitative measure of the rate constants for interaction of the protein with the substrate [15]. As Peracchi put it elegantly [16]: “Substrate specificity cannot be absolute and is inherently limited. … discrimination between alternative substrates can be relatively low, … Substrate promiscuity helps to fuel an ‘underground’ network of reactions which may represent a basis for further evolution and diversification of metabolism”. Notably, binding protein selectivity is tested with only few analytes, while the in vivo environment presents a highly complex set of molecules. Rarely, binding protein affinity is suitable for in vivo analyses. Affinity has to be adjusted by mutagenesis and affinity series of the sensors might be required. Mutations in the binding pocket may impact ligand selectivity.
(5) Cytotoxicity and biostability. From first principles, one could argue that the higher the sensor level is, the brighter the signal and the better the ability to discern changes in analyte levels or activity. Strong promoters provide high levels, yet besides likely triggering gene silencing, high sensor levels impact physiology. While sensors are minimally invasive, they can affect cellular functions, either by acting as scavengers or by interacting with other cellular components; essentially posing an “Observer Effect” problem. In the absence of novel, even less invasive technologies, it will be important perform proper controls. Biostability of the biosensor is a very important characteristic especially for biosensors used for continuous monitoring. This feature determines the ability of the biosensor device to resist change in its performance over a period of time in response to interruptions arising from external factors.

## 4. Biomedical Applications

From the beginning in the early 2000s, genetically encoded fluorescent biosensors have conducted numerous insights into the biochemical pathways of a cell. In the last decade, genetically encoded fluorescent biosensors attracted the increasing interest of researchers working in the field of biomedicine to gain data on pathological processes being emerged under the exposure of hazardous agents or physiological disturbance. From the single cell being cultured in vitro to the whole tissue, biosensors provide direct measurements of the dynamics of macromolecules activities, fluctuations in physical and chemical parameters (e.g., ions flow or pH changes) and enable registration drugs potency and their effects on the target pathways to improve drug development and increase the efficiency of medical care.

### 4.1. Cancer

A large number of biosensors have been designed and applied to study cancer cells and tumor tissues. Cancer is a large group of diseases characterized by abnormal cell growth, the ability to diffuse into the different tissues of the body, and tumors formation. Moreover, the disease is known to be highly heterogeneous in both intertumoral and intratumoral levels. Cancer tumors keep developing after the malignant transformation, and the cells undergo different exposure, leading to the genetic heterogeneity and/or variety in molecular pathways in the cells within one tumor type. This feature of cancer complicates its research and drug development, as pathogenic mechanisms are not identical even for cells belonging to the same tumor [17,18,19]. Thus, it opens the great perspectives for genetically encoded fluorescent biosensors application for investigation of the pathological processes and anti-cancer drugs screening. Many intrinsic pathways accompany tumor formation, such as mutations in transcription factors or tumor-associated antigens presentation. Genetically encoded fluorescent biosensors are supposed to be an appropriate toolkit for these molecular approaches, as biosensors can be integrated into any metabolic pathway of a cell.

#### 4.1.1. Protein Kinase Activity

**Figure 3:**
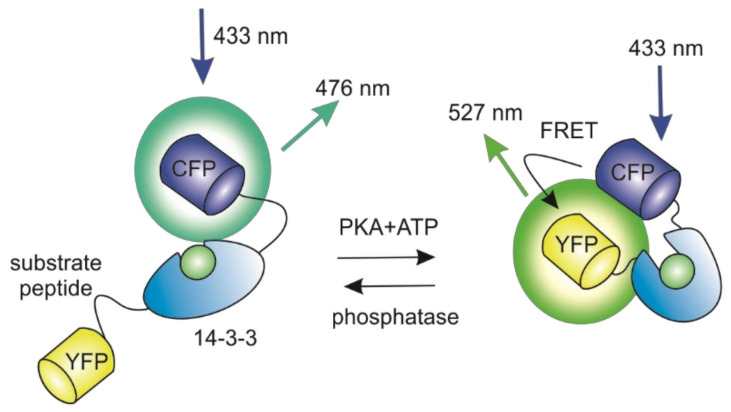
Schematic representation of ratiometric indicator for visualizing protein phosphorylation.

Many intrinsic pathways accompany tumor formation, such as mutations in transcription factors or tumor-associated antigens presentation. Genetically encoded fluorescent biosensors are supposed to be an appropriate toolkit for these molecular approaches, as biosensors can be integrated into any metabolic pathway of a cell. As a basis of cancer formation, living cells in the proliferation and aging process accumulate genetic mutations, which as a result can lead to the inactivation of tumor suppressor genes. Thus, protein kinase (PK) signaling is one of the central networks involved in malignization. The network regulates cellular proliferation and cell survival, so the mutations occurring in the genes of the protein members of this pathway can affect cellular malignancy. Therefore, their observation is an area of great interest for the investigation of cancer formation. The first experiment in the field of protein kinase study with genetically encoded fluorescent biosensors was performed in 2001 by a group of Jin Zhang. In the research, the scientists aimed at cAMP-dependent protein kinase A (PKA) and presented a FRET-based biosensor AKAR constructed from CFP fused with a phosphoramide acid-binding domain (14-3-3) and YFP (Figure 3). The domain is enabled to specifically detect and bind the phosphorylated peptide. The increased level of cAMP leads to phosphorylation alterations resulting in changes in the emission of two fluorophores. Phosphorylation increases intramolecular binding between 14-3-3 and the substrate, and dephosphorylation reverses this effect. As the kinase activity usually changes against a relatively stable phosphatase activity, the resulting FRET changes between the pair of CFP and YFP allow this construct to reflect fluctuations in kinase activity [20].

However, the later versions of biosensors were based not on the 14-3-3 domain, but on an FHA1 domain that has a lower affinity and does not lead to irreversible stress-response. Also, in 2011, a group of scientists developed a bioluminescent sensor for protein kinase A and protein kinase C (PKC) activities. The reporter consists of both luminescent and FRET reporter units and is able to switch according to the kinase activity. Thus, the increased PKA activity recovers the catalytic activity of the luminescent part that leads to luminescence [21]. Later, the FRET-based biosensors were modified for FLIM technology. A Picchu-FLIM sensor was generated based on the FRET EGFR activity biosensor phosphorylation indicator of the CrkII chimeric unit [22] by replacing YFP with mRFP1 and CFP with EGFP [23]. The expanded data of kinases reporters are presented in Table 1.

As protein kinase activities impairments are central in a wide variety of cancer cases, their behavior as biomarkers of cancer development or pharmacological targets is of particular interest. Genetically encoded fluorescent biosensors utilization enables quantifying the kinase activities and further detecting pathological dysregulations and selecting therapeutic drugs. Moreover, biosensors provide reliable data on drugs resistance and specific cancer markers status during disease development [38]. Thus, there were developed FRET-based sensors to assess the efficiency of drug therapy in patients with chronic myeloid leukemia. The sensors allow the estimation of the effect of imatinib and kinase inhibitors on BCR-ABL activity in single cells and predict the most efficient drug for a patient [39].

#### 4.1.2. pH Level

In addition, an increased intracellular pH rate triggers the metabolic switch of the cancer cells from normal oxidative phosphorylation to aerobic glycolysis, also known as the Warburg effect. During the pathological process, cells produce ATP energy primarily via active glycolysis followed by lactic acid formation [40]. On this basis, the Warburg effect is one of the hallmarks of malignization, and a biosensor focused on lactate transporter activity and production can be used to provide data on cancer cell progression. Thus, based on the bacterial transcriptional regulator LldR as a recognition element, San Martin et al. generated a “Laconic” FRET biosensor that provides acute lactate detection in the physiological range between 1 uM and 10 mM [7]. Also, pyruvate as the terminal product of glycolysis can be turned into lactate by lactate dehydrogenase or transported into the mitochondria for subsequent ATP generation. Consequently, pyruvate carrier activity monitoring can be a promising approach for Warburg effect detection. So, the BRET-biosensor RESPYR based on MPC (mitochondrial pyruvate carrier) was developed by Compan et al. In the article, the authors revealed that pyruvate level elevating in a couple with monocarboxylate transporter inhibition could be one possible strategy to improve MPC activity and reverse the Warburg effect [41].

#### 4.1.3. Other

Decreased p53 activity is associated with tumorigenesis and cancer progression. Furthermore, p53 is ubiquitinylated and targeted to degradation by hDM2 protein, which is overexpressed in some types of cancer [42]. In order to find novel p53-hDM2 interaction inhibitors, a system for automated high-content screening for protein-protein interaction disruptor was established. First, p53 was fused to GFP and augmented by an NLS localizing the construction in the nucleolus. hDM2 carried both NLS and NES and was fused to RFP. Both proteins colocalized in the nucleolus. In the presence of the known p53-hDM2 interaction inhibitor Nutlin-3, hDM2 was exported to the cytoplasm. A library of 220,000 small-molecule compounds was screened, and the assay demonstrated high reproducibility. Finally, three compounds related to methylbenzonaphthyridin-5-amine were confirmed to increase p53 protein level and apoptosis and to cause cell cycle arrest and growth inhibition in a p53-dependent fashion [43].

The activation of effector caspase-3 is one of the most critical steps of apoptosis [44]. A FRET-based reporter of caspase-3 activity has been created. It consists of CFP, YFP, and a linker sequence containing the caspase-3 recognition site. After caspase-3 activation caused by apoptotic stimuli, the sensor protein is cleaved, and the FRET ratio decreases. FRET ratio does not decrease in necrotic cell death. When expressed in HeLa-C3 cells grown in microplates, the sensor was able to dose-dependently reflect the pro-apoptotic effect for several compounds of known biological activity, such as vincristine, paclitaxel, and hydroxyurea, as well as for some novel plant-derived substances [45].

Immunogenic cell death inducers stimulate cancer cells to emit signals that attract and activate immune cells. During premortem stress several processes occur, for example exposure of calreticulin on the surface of the cell, release of ATP, and the exodus of high-mobility group box 1 (HMGB1) protein from the nucleus to the cytoplasm [46]. LC3 protein migrates to autophagy-specific granules [47]. Using U2OS human osteosarcoma cells expressing calreticulin fused to RFP and HMGB1 or LC3 fused to GFP, a library of more than 500 compounds was screened. This screening demonstrated that some tyrosine kinase inhibitors can induce immunogenic cell death, which was further confirmed in cell cultures and murine xenografts [48].

### 4.2. Neurological Disorders

**Figure 4:**
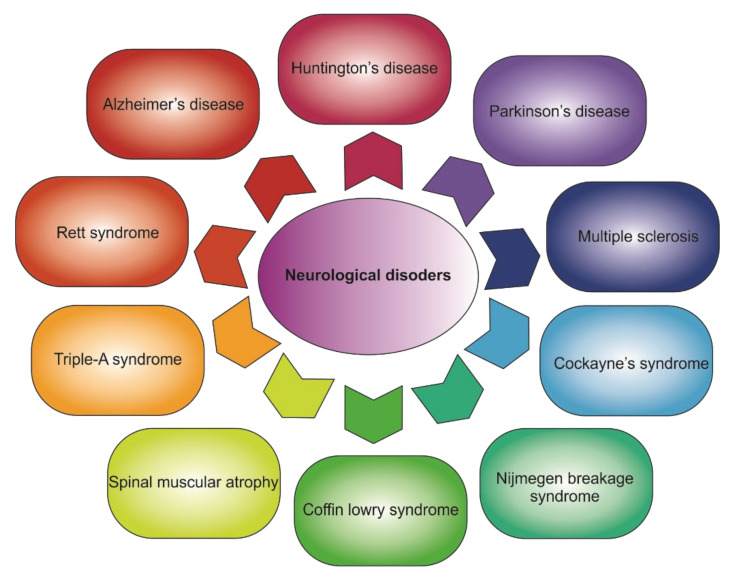
Common neurological disorders.

A group of neurological disorders includes any nervous system disorder: from nerve injury resulting from physical trauma to genetic neurodegenerative processes (Figure 4). Referring to the biological causes of diseases, abnormalities of neural tissue development are caused by processes such as specific protein accumulations in the matrix or errors of posttranslational modification. Today, the majority of the molecular processes occurring during the pathological process remain unclear, as well as the ways to prevent neurological disorders and their treatment [49]. Neurological disorders vary in several common characteristics and fundamental processes leading to neuronal dysfunction. Remarkably, there are no available in vivo biomarkers for disease diagnosis today other than reverse genetics methods [50]. Given this, it is of particular interest to investigate different molecular pathways in living systems in real time. Genetically encoded fluorescent biosensors meet these requirements and can be targeted to visualize various metabolic and signaling pathways of the cell and elucidate the origin of both pathological processes in disease and the drugs’ effect on defect neurons.

#### 4.2.1. Measurements of Lactate Level

Tissues under hypoxia release large amounts of lactate. Earlier, lactate overproduction was supposed to be a pathogenic process during hypoxia, however, today it is clear that lactate plays a significant role in normal oxygenated cells as its level significantly rises during neuronal activity [51]. Lactate is involved in the myelinization processes and acts as an intracellular signal in neurovascular coupling and sodium sensing [52]. Thus, previously described a “Laconic” enables lactate detection in the physiological range and can be transduced into targeted cells for in vivo measurements of lactate [7].

The in vivo and in vitro imaging in cortical astrocytes and neurons via Laconic was performed by Machler et al. [53]. It is known that lactate transport through the membrane is accomplished via MCTs (monocarboxylate transporters), and the conversion between lactate and pyruvate is catalyzed by LDH (lactate dehydrogenase). The differences between lactate affinity of MCTs and LDH in neurons and astrocytes provide an astrocytic production and neuronal consumption of lactate [54]. In the study, Machler et al. firstly showed lactate gradient from astrocytes to neurons on mice models in vivo [53]. The obtained data may aid the drug screening upon the epilepsy treatment as the LDH inhibition increase the epileptic activity of neurons [55] as well as understand the mechanism of Huntington’s disease development [56]. Also, the drug screening via Laconic sensor can be utilized to detect mitochondrial impairments under toxicants exposure [57].

#### 4.2.2. pH Level

As it was mentioned before, under normal conditions, intracellular pH (ipH) is lower than extracellular pH (epH). The pH value is one of the most significant physiological parameters of a cell that impact on neural development, cell division, migration, apoptosis, etc [58]. Within the nervous system, pH activates neural functions whereas activated neurons can generate shifts in intracellular pH. Furthermore, H+ export, as it has been shown in *Xenopus laevis*, is supposed to be crucial for cell regeneration [59]. The genetically encoded reporters have a great potential to monitor H+ rate at both cellular and subcellular levels and can be utilized to elucidate a mechanism of neural disorders, as well as bring us closer to neural regeneration improvement.

The familiar H_2_O_2_-sensitive sensor HyPer2 [60] gave rise to a new pH sensor SypHer2, which was applied in neuroscience and regenerative biology [61]. The SypHer2 demonstrated improved brightness compared to the previous sensor SypHer [62] and was applied to observe the pH activity upon the blockade of GABA-receptors as a model of epileptiform activity. Also, the experiments on amputated tails of *Xenopus laevis* revealed the appearance of pH gradient with the great acidification in cells adjacent to the wound. The data may suggest that pH drop can influence the regeneration. However, the brightness of SypHer and SypHer2 was not optimal for a high spatio-temporal resolution performance. Thus, a new SypHer-based sensor SypHer3s bearing mutations Y145F, D129G in the GFP part presented increased brightness and an elevated dynamic pH range [63]. As the reporter capable of detecting fluctuations of pH in cells and cellular compartments, it has great potential for neuronal in vivo and in vitro measurements [64,65].

Not only ipH and epH impact on the development of neurological disease but even lysosomal pH. Lysosomes are involved in the autophagy-lysosome pathway of misfolded protein elimination and require acidic pH (4.5–4.7) to maintain their functions [66]. The lysosomal pH depends on the vacuolar-type H+ -ATPase (V-ATPase) proton pump which create pH gradient by proton translocation and ATP hydrolysis [67]. Alkalization or acidification of lysosomal pH can trigger numerous disorders of nervous system from dementia [68] to amyotrophic lateral sclerosis [69]. Nowadays, chemical dyes such as LysoTracker are the most commonly used methods of pH detection, however, in recent years genetically encoded reporters for lysosome pH measuring have appeared. For example, Ponsford presented a ratiometric sensor RpH-LAMP1-3xFLAG, which was based on widely expressed lysosomal protein LAMP1. In the study, three drug treatments for lysosomal pH change were examined: chloroquine, apilimod, and torin2. The data suggest that chloroquine and apilimod treatment increased the lysosomal size and inhibited the activity of the MTORC1 complex, which response to alkalization is still under investigation. At the same time, these drugs had a significant effect on lysosomal pH, whereas the effect of torine2 did not have any impact [70]. Also, Chin et al. constructed a FIRE-pHLy sensor targeted to the same LAMP1 protein. The utility of the sensor was observed in induced neurons derived from induced pluripotent stem cells as well as neuroblastoma cells. The study shows that the FIRE-pHLy can be applied for lysosomal pH detection in various neurodegenerative disease cell models. It may be suggested that the new reporter is a novel promising tool to profile lysosomal pH dynamics in cellular neuron systems, and the sensor can be applied in lysosome-based drug discovery in future [71].

#### 4.2.3. Other

Despite the numerous research of neurological disorders development and their progression, the precise molecular pathways and targets for drug treatment remain unclear. The genetically encoded tools in combination with specific cell types can be integrated into every molecular pathway to visualize the interplay of molecules during disease development. Hence, multiple sensors are aimed at widespread neuromodulator dopamine. Despite dopamine being a central transmitter in a variety of pathological and physiological processes (schizophrenia, attention deficit hyperactivity disorder, Parkinson’s disease), monitoring of dopamine changes in live animals is still a problem. Nowadays, sensitive readouts of dopamine have been developed for various animal models. Thus, dLight1 sensor provide detection of submicromolar dopamine concentration changes at dendrites and single dendritic spines in mice [72] whereas GRABDA sensor have an optimized brightnesses in experiments not only in mice, but in zebrafish and flies [73]. For more information, the recent advances in dopamine sensors were perfectly described in recent reviews [74,75].

Besides neurotransmitters, metabolites of the cell also play a critical role in neurodegeneration. For example, glucose, as the energy source of brain, can be detected by multiple reporters [76,77,78,79,80]. The obtained data via sensors allowed estimation of an intracellular glucose concentration in the brain of ~1.4 mM. Also, Suzan Youssef with a group decided on the role of another cell metabolite, hydrogen sulfide (H2S), during neurodegenerative disease development [81]. H2S in organism releases during sulfur metabolism and acts like a gasotransmitter [82], and its dysregulation is related to several neurological disorders [83]. Thus, a novel ratiometric biosensor hsFRET was designed for sensitive monitoring of H2S dynamics in real time and validated in HEK293T. As a result, the study demonstrated that hsFRET can be applied for in vitro experiments in mammalian cells [81].

### 4.3. Inflammation

**Figure 5:**
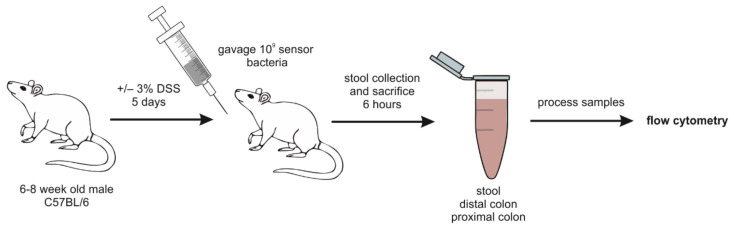
In vivo measurement of thiosulfate and tetrathionate in healthy and inflamed mice. Experimental design. 6- to 8-week-old C57BL/6 mice were given water with or without 3% DSS for 5 days before oral gavage with sensor bacteria. After 6 h, samples were collected from the mice, processed, and analyzed by flow cytometry to measure GFP production.

Numerous genetically encoded fluorescent biosensors were created to screen gut inflammation both for in vitro [84] and in vivo [85] models. In contrast to the previous reporters, based on mammalian cells and aimed to elucidate the processes of the organism the cells referred to, sensors of gut inflammation involve prokaryotic microbiota. For example, a biosensor described by Kristina N-M Daeffler presented a gut-adapted *E. coli* strain with insertion of elements from *Shewanella* sp., sensitive to hydrogen sulfide (H2S) metabolites thiosulfate and tetrathionate [86]. The mice were fed engineered bacteria, and their feces were analyzed by flow cytometry (Figure 5). This study showed that intestinal inflammation activates the reporter, so the level of H2S metabolites may reflect colitis. Another designed sensor based on *E. coli* responds to increased nitric oxide level [87] that is known to be a biomarker of gut inflammation [88]. The system was validated in the murine model in vivo and can respond to physiological nitrate levels in the gut. Moreover, the authors propose to replace the reporter gene with an anti-inflammatory gene to create a therapeutic device for colitis.

As most biomarkers of intestinal inflammation are under investigation, one promising approach in synthetic biology involves the use of biosensors aimed at detecting several signaling molecules of the pathological process within a single experiment. Thus, researchers from Harvard Medical school created an *E. coli* strain with a genetic memory element based on lambda cI/Cro system [89] and then modified it with a transcriptional promoter activated in the presence of a certain stimulus [90]. Based on the data of cell responses, it is possible to build biosensor trigger libraries that can predict the potential markers of intestinal inflammation. The created system allows the detection of pathological pathways that cannot be constructed in a rational way with the existing knowledge about colitis biomarkers.

Not only the inflammation of the entire organ but even inflamed tissue can be detected by bacterial-based fluorescent sensors. For example, a strain of *E. coli* with GFP expression, which induced an immune response after injection into the ear of mice, responded to the resulting inflammation by increasing fluorescence [91].

### 4.4. Other Diseases

One of the major reasons for mortality throughout the world is cardiovascular diseases. Voltage-gated ion channels play crucial roles in many cellular processes and are particularly important for the functioning of electrically excitable cells such as cardiomyocytes. This places them among the key drug targets for cardiovascular disorders and is why the development of high throughput screening methods for voltage-gated ion channel inhibitors and modulators is important. The development of genetically encoded voltage indicators and optogenetic actuators opened opportunities for all-optical electrophysiology [92,93]. The all-optical Optopatch approach was utilized in one of the early attempts to develop a cardiotoxicity assay [94]. In this screening concept two populations of human induced pluripotent stem cell derived cardiomyocytes were cultured together in a mixture: one subset expressed CheRiff actuator [95], and the other expressed the fusion protein CaViar (Ca^2+^ and Voltage indicator [96]). CheRiff-expressing cells were used for pacing the entire syncytium of cardiomyocytes by blue light pulses, while CaViar allowed for simultaneous recording of the resulting membrane potential and Ca^2+^ fluctuations in the other cardiomyocytes population. Although not tested in real screening, Cardiac Optopatch has shown good performance in testing compounds with known mechanisms. It successfully demonstrated the effects of drugs on action potential waveforms and Ca^2+^ dynamics in spontaneously beating cultures and cultures paced at different frequencies. Moreover, the screening platform was proven to be suitable for studying long-term drug effects, which may allow it to be used in delayed drug cardiotoxicity assays.

The alternative mixed electrical-optical approach employing a combination of field stimulation of human induced pluripotent stem cell-derived cardiomyocytes with genetically encoded voltage indicators recording has also demonstrated its applicability for cardiac drug evaluations. VSFP-CR, a FRET-based voltage sensor consisting of the voltage sensing domain of a potassium channel and a GFP/RFP FRET pair [97,98] was used for cardiomyocyte subtype-specific action potential imaging [99]. Placing the sensor under the control of lineage-specific cardiomyocyte promoters made it possible to detect and measure the changes in action potential duration and the occurrence of early afterdepolarizations caused by deleterious mutation or induced by drugs in patient-specific human induced pluripotent stem cell-derived ventricular-, atrial-, or nodal-like cardiomyocytes.

ArcLight A242 [100], a variant of Ciona intestinalis voltage-sensing phosphatase-based sensors containing fluorescent protein super ecliptic pHluorin, was utilized in another series of experiments [101,102]. ArcLight-expressing human induced pluripotent stem cell-derived cardiac cell sheets were used for optically mapping the electrical activity in a two-dimensional cardiac tissue model during different experimental conditions, including electrically- and drug-induced arrhythmias and arrhythmia-preventing interventions [103].

**Figure 6:**
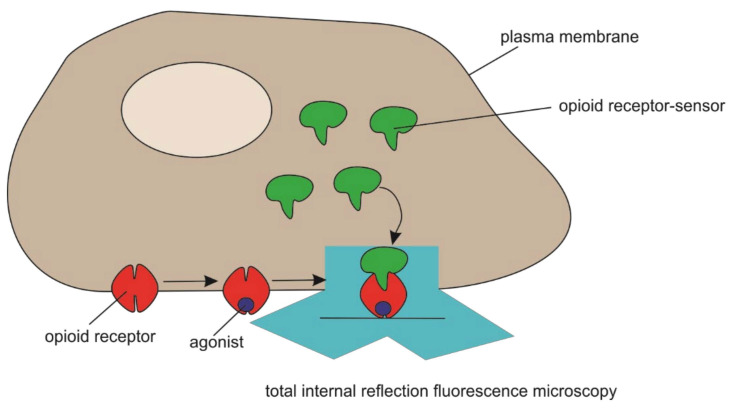
Schematic of opioid receptor-sensor and mu-opioid receptor localization in cells and expected opioid receptor-sensor re-localization upon agonist addition. Total internal reflection fluorescence microscopy light beam is indicated.

Opiate alkaloid drugs, such as morphine, are among the most effective agents known for alleviating pain. However, such drugs produce significant toxicity and have high abuse potential. These factors have contributed to opioid addiction becoming a large and growing public health problem globally [104]. Stoeber et al. described the biosensor derived from a conformation-specific nanobody that is capable of detecting ligand-induced activation of mu- and delta-opioid receptors in living neurons (Figure 6) and demonstrated that this conformational biosensor provides precise spatial and temporal resolution of opioid receptor activation and deactivation in situ with minimal perturbation of function [105]. The authors provided functional evidence supporting the hypothesis that internal opioid receptor activation contributes to the cellular signaling response. These results reveal a characteristic pattern of subcellular opioid receptor activation generated by peptides and its profound distortion by drugs.

Nicotine dependence is thought to arise in part because nicotine permeates into the endoplasmic reticulum (ER), where it binds to nicotinic receptors (nAChRs) and begins an “inside-out” pathway that leads to up-regulation of nAChRs on the plasma membrane. However, the dynamics of nicotine entry into the ER are unquantified. Targeting genetically encoded fluorescent biosensors for nicotine, termed iNicSnFRs, to the plasma membrane or to the ER and measuring nicotine kinetics in HeLa, SH-SY5Y, N2a, and HEK293 cell lines, as well as mouse hippocampal neurons and human stem cell–derived dopaminergic neurons, made it possible to run combined pharmacokinetic and pharmacodynamics simulations of human smoking [106]. iNicSnFRs enable optical subcellular pharmacokinetics for nicotine and the smoking cessation drug varenicline during an early event in the inside-out pathway.

## 5. Advantages, Challenges, and Prospects for the Use of Genetically Encoded Fluorescent Biosensors in Biomedical Research

Genetically encoded fluorescent biosensors have emerged as promising alternatives to traditional conventional techniques over the past about five decades. Genetic manipulation of the cell genome has opened opportunities to visualize and study molecular interactions in their native environment. At the same time, improved cell culture methods and emergence of new 3D culture matrices can lead to the integration of cell culture with electronic devices. The study of human induced pluripotent stem cells has been one of the main directions in biology in recent decades. The properties of pluripotent cells provide abundant opportunities for their using in therapy and disease modeling, as well as in the new drugs search and test. Currently, there are many well-developed methods for obtaining various types of differentiated cells from human induced pluripotent stem cells; researchers developed technologies for creating organoids and tissues based on various induced pluripotent stem cell derivatives. An enormous variety of cell models of human diseases, including neurodegenerative diseases such as Alzheimer’s disease, Parkinson’s disease, and other diseases, have been obtained based on induced pluripotent stem cell and their differentiated derivatives. The use of directed genome editing technology, as well as the use of chemical or genetically encoded biosensors in cell models, provides new knowledge about normal and pathological processes in living cells. However, the use of chemical biosensors has its drawbacks. There are commercial, chemically synthesized molecules that are not renewable and therefore must be purchased continuously. In addition, there is a problem with the delivery of chemical dyes to exact cellular compartments, which are solved by various modifications of the biosensor molecule. Complex modifications increase the size of the biosensor molecule, change its physicochemical characteristics, which prevents its passage through the cell mem-brane. Another important point when using chemical biosensors is that the number of biosensor molecules entering the cell is unlimited. Therefore, if there is a correlation between the response and the biosensor concentration, the measurements obtained will not correspond to the actual picture occurring in the cell.

Genetically encoded biosensors can be inserted into safe-harbor loci, for example, the *AAVS1* locus. It provides a strictly fixed number of transgene copies per genome, and its expression presumably occurs without disturbing the expression of adjacent or more distant genes. The use of an inducible promoter provides controlled expression, allowing the elimination of side and off-target effects. Using various combinations of genetically encoded biosensors embedded in several safe-harbor loci, or combining chemical biosensors with genetically encoded ones, one can thoroughly investigate the same process occurring in a cell. Today, the field of dynamic measurements of intracellular processes is developing very rapidly, and the leading task for the near future is the widespread use of the described technologies and methods not only in individual cells but also in more complex systems, such as cell organelles and whole organisms. The research of the mechanisms of complex biological processes, such as embryogenesis and aging, inflammation and regeneration, the functioning of tissues and organs in normal conditions and during the development of pathology, symbiotic interactions of organisms, the interplay between the host and the pathogen, and many others, require in vivo models.

Low fluorescence intensity or small response amplitudes of biosensors can be a practical limitation for in vivo use. Registration of subtle physiological changes in tissues with such instruments can become difficult. It is especially true for in vivo studies on mammalian models. As a rule, in these systems, the signal must be recorded in deep tissue structures, which requires special optical equipment.

Regardless of the biosensor used, it is important to remember that the introduction of an exogenous protein into the cell or organism can lead to unexpected effects that have an impact on physiological processes. Although it is generally accepted that fluorescent proteins are inert reporters, there are examples of their negative influence on intracellular processes in the literature [107].

Despite the above-mentioned possible limitations, biosensors have indisputable advantages over many other methods; they are gaining widespread popularity and are actively used for solving various questions, including drug screening, optimization, toxicity, or mechanism of action studies [105,108,109]. The development of biosensors for the detection of new compounds is a crucial line of future research that will open new perspectives for its application in experimental models [110,111,112].

A separate issue in the context of imaging with biosensors arises from the fact that shifts in the protein concentration or the thickness of the biological sample can result in signal alterations that might be taken for changes of the specific parameters. The described problem is especially relevant when imaging with single fluorescent protein-based sen-sors; however, FRET indicators are also prone to many artifacts. Common fluorescent proteins demonstrate relatively broad spectra, which results in tangible bleedthrough making interpretation of ratiometric signal difficult. Moreover, ratiometric readout faces challenges in the case of confocal microscopy. Depending on the depth of the sample, patterns of light scattering for emission channels can differ notably leading to measurement artifacts, especially in the case of in vivo imaging. A solution might be found in the implementation of FLIM readout [113]. The main advantage of this approach is that fluorescence lifetime is a pure physical parameter independent of chromophore concentration, photobleaching, and the settings of equipment (intensity of excitation light and optical path).

Insufficient transparency of tissues for visible light due to various factors including melanin and hemoglobin absorbance and relatively pronounced scattering hamper im-aging of multicellular organisms. Moreover, long microscopic series lead to a decrease in fluorescent biosensors brightness due to photobleaching. The widefield microscopy more enhances this effect. Multiphoton microscopy methods can partially overcome these problems. This approach is based on simultaneous excitation of a chromophore by several photons with wavelengths, which are longer than that for the emission maximum. Multiphoton microscopy also allows to shift of the source of excitation to the infrared region, which facilitates imaging of deep tissue regions. As multiphoton absorption is characterized by low efficiency, this approach requires focusing the laser at a small sample volume, which reduces photobleaching and improves the signal-to-noise ratio. The improvement of biosensors, as well as the approaches for their visualization inside living organisms, will provide further progress for in vivo biomedical studies.

## 6. Conclusions

Genetically encoded fluorescent biosensors are novel tools for biochemical, cytological, and physiological research, especially in the field of biomedicine. They allow real-time detection with a high spatio-temporal resolution of numerous pathways within the live cell, both in vitro and in vivo. As a significant advantage, sensors can be integrated into different model systems, from 2D cell cultures to an entire organism. Nowadays, biosensors clarify the basis of diverse diseases such as cancer, neurodegeneration, inflammation, etc., and have become a promising approach for drug screening.

Despite the benefits, the use of genetically encoded fluorescent biosensors is currently in its infancy. However, their potential opens great opportunities for personalized medicine. Today, cell-based sensors can be implemented for targeted therapy of allergy via the modification of a patient’s blood cells, which can sense markers of allergy reaction and respond through its inhibition [114,115]. Moreover, sensors can cure psoriasis through sensing TNF-a and IL-22 as biomarkers of psoriasis with the following expression of the therapeutic cytokines IL-4 and IL-10 [116].

Overall, the application of genetically encoded fluorescent biosensors seems to have a great future since there are many promising directions in improving sensor sensitivity and developing new model systems. A wide range of experiments aims at improving imaging techniques and developing new model systems both for elucidating protein interactions and for drug screening and personalized therapy.

## Figures and Tables

**Table 1:** Genetically encoded fluorescent biosensors for protein kinase studying in cancer.

Kinase	Name	Type of Sensors	Proteins	Reference
Protein kinase A	AKAR	FRET	ECFP/YFP	[20]
	AKAR2	FRET	ECFP/Citrine	[24]
	AKAR3	FRET	YPet	[25]
	*Rluc*-PCA	Bioluminescence	*Renilla* luciferase	[26]
	FLIM-AKAR	FLIM–FRET	meGFPΔ/cpsREACH	[27]
	ExRai-AKAR	Ratiometric	EGFP	[28]
	BimAKAR	FRET	YPet	[21]
Protein kinase A/Extracellular signal-regulated kinase	ERK/PKA biosensors	FLIM–FRET	sREAChet/EGFP	[29]
Protein kinase B	*Akt*AR	FRET	CFP/Venus	[30]
Protein kinase C	BimCKAR	Bioluminescence	*Renilla* luciferase	[21]
	CKAR	FRET	CFP/YFP	[31]
Protein tyrosine kinases Src, Abl	Src/Abl indicator	FRET	CFP/YFP	[32]
Protein tyrosine kinase Src	Src reporterr	FRET	CFP/YFP	[33]
BCR-ABL kinase	Pickles	FRET	CFP/Venus	[34]
Serine/threonine protein kinase	TORCAR	FRET	Cerulean/YPet	[35]
LATS kinase	LATS-BS	Bioluminescence	*Photinus Pyralis* luciferase	[36]
P38 MAP kinase	PerKy-38	FRET	YPet/CFP	[37]

## Data Availability

Not applicable.
